# Interventions in Maternal Anaemia to Reduce Maternal Mortality Rate Across India

**DOI:** 10.7759/cureus.46617

**Published:** 2023-10-07

**Authors:** Manisha Totade, Abhay Gaidhane, Palash Sahu

**Affiliations:** 1 Community Medicine, Jawaharlal Nehru Medical College, Datta Meghe Institute of Higher Education & Research (Deemed to be University), Wardha, IND; 2 School of Epidemiology and Public Health, Jawaharlal Nehru Medical College, Datta Meghe Institute of Higher Education & Research (Deemed to be University), Wardha, IND

**Keywords:** pregnancy, oral iron therapy, anaemia mukt bharat, iron deficiency anaemia, maternal mortality

## Abstract

Anaemia is one of the most prevalent issues encountered throughout pregnancy, with Iron deficiency anaemia and megaloblastic anaemia being the most common causes in India. It is critical to address anaemia in pregnancy since it has been linked to adverse pregnancy outcomes like preterm delivery, low-birth-weight newborns, fetal mortality, and, in certain circumstances, maternal death. The maternal mortality rate (MMR) is one of the significant health challenges, particularly in developing countries. It has substantially impacted the population's social situation and requires quick management. In this review article, we discuss recent developments and advancements in treating maternal anaemia with the aid of some government health programs, which can help with lowering the risk of maternal mortality. The primary goal of this manuscript is to raise awareness about anaemia in pregnancy. We examined the literature on anaemia during pregnancy, with a view to offering current and unambiguous guidance for preventing and managing this illness, which, if not appropriately managed, can result in severe maternal and neonatal problems.

## Introduction and background

Anaemia [Haemoglobin (Hb) levels below 6 gm/dl] has been associated with detrimental pregnancy outcomes. Severe maternal anaemia can lead to low-birth-weight babies, premature deliveries, spontaneous abortions, and fetal deaths. However, fetal Hb concentration appears unaffected by mild to severe iron deficiency. Lower limits for Hb values in the first trimester are 11 g/dl, while they are 10 g/dl in the second and last trimesters [1]. Anaemia primarily stems from various factors, including inadequate nutrition, insufficiency of iron, deficits in essential micronutrients like folic acid, vitamin B12, and vitamin A, as well as underlying health conditions such as malaria, hookworm infestations, and schistosomiasis. Furthermore, anaemia can be linked to factors like HIV infection as well as genetically inherited Hb disorders like thalassemia [2]. During pregnancy, natural physiological changes alter Hb, causing a relative or quantitative drop in Hb level. Iron deficiency anaemia (about 75% of cases) and megaloblastic anaemia (roughly 25% of cases) are the most commonly observed anaemias during pregnancy, and they are more common in women who consume poor diets and fail to take prenatal iron and folate supplements [3].

Iron deficiency is the most common nutritional deficit worldwide, accounting for 75% of anaemia cases during gestation. The incidence of anaemia in pregnancy varies substantially among countries due to differences in socioeconomic circumstances, ways of life, and healthcare-seeking practices. Anaemia affects pregnant women all over the globe (the worldwide prevalence in pregnancy is estimated to be approximately 41.8%), with incidence rates ranging from 35 to 60% in Africa, Asia and Latin America, and 20% in nations with advanced economies [4]. Anaemia during pregnancy affects more than 80% of the world's population [5]. The estimated prevalence of anaemia in the United States is 5.7%, while it is 75% in Gambia and 65-75% in India [6]. Improving healthcare settings, particularly in rural areas, is of utmost importance. It involves increasing the accessibility of healthcare services, ensuring the availability of educated healthcare personnel, and enhancing access to diagnostic instruments. Many countries have established strong monitoring and surveillance systems that aid in tracking the incidence of anaemia, shedding light on high-risk areas, and assessing the efficacy of treatments.

India has the highest maternal mortality rate (MMR) compared to any other country, accounting for nearly a quarter of all pregnancy and delivery-related maternal deaths globally [7]. According to an Indian hospital research, the MMR is 4.21/1000 live births. Conditions such as haemorrhage, hypertensive diseases, infection, ruptured uterus, hepatitis, and anaemia cause 50-98% of maternal mortality. Illegally induced abortions cause half of all maternal deaths from sepsis. MMR has not sufficiently declined in India during the previous 15 years. Age, elderly primigravida, unexpected pregnancy, grand multipara and associated unsafe abortion are the factors usually associated with MMR [8]. At the national and subnational levels, obstetric haemorrhage is the principal cause of maternal fatality. Chronic underlying illnesses, as well as delayed diagnosis and medical treatment, may exacerbate obstetric haemorrhage [9].

MMR has decreased as a result of effective government and stakeholder programmes like the Child Survival and Safe Motherhood Programme (1992), the Reproductive and Child Health Programme (1997), and the National Health Mission (2005) [10]. Effective maternal anaemia therapies directly influence MMR in India. Maternal anaemia is linked to pregnancy and delivery issues such as bleeding and infections. The likelihood of these consequences is considerably lowered when anaemia is properly treated. The quality of emergency obstetric therapy has a significant impact on maternal mortality [11]. Advances in institutional deliveries, as well as community-based interventions that result in fewer maternal fatalities due to direct causes, should be maintained. However, to reduce maternal deaths even more significantly, focusing on treating indirect reasons for maternal mortality during pregnancy at the community and hospital levels is very vital [12].

## Review

Methodology

**Figure 1:**
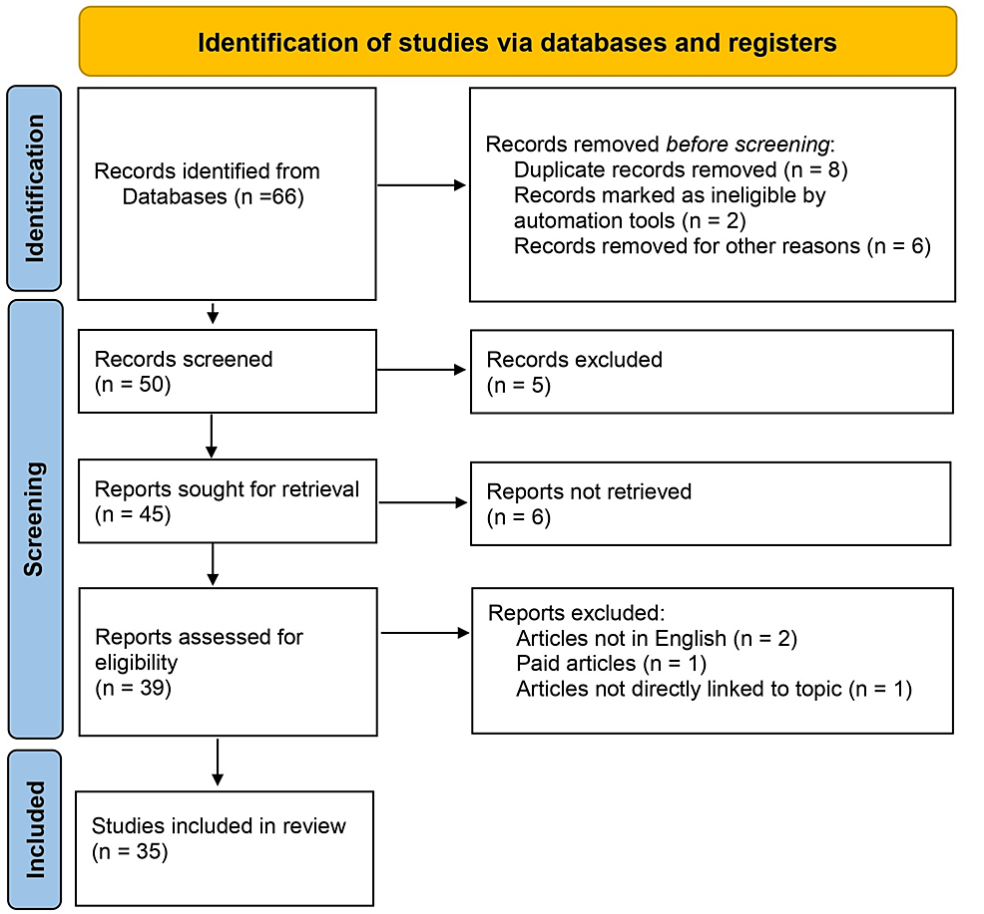
The PRISMA flowchart depicting the study selection process PRISMA: Preferred Reporting Items for Systematic Reviews and Meta-Analyses

The keywords "maternal mortality," "anaemia in pregnancy," "interventions in maternal anaemia," "PPH," and related synonyms were used to conduct a search for relevant studies in English on the online databases PubMed and Google Scholar. With no specific date limit, the search encompassed publications published from the databases' creation. This ensured that only the most recent research on the topic would be included in the review. We aimed to include peer-reviewed papers published in English that focus on treatments for maternal anaemia to reduce the maternal death rate in India; paid articles, articles not in English, and articles not directly linked to the topic were excluded. The initial screening involved reading the titles and abstracts of the selected publications with a view to applying the inclusion criteria. Full-text publications of potentially relevant research were collected, and the final articles for the review were chosen based on further screening. Ultimately, 35 articles met our criteria and were included in the final analysis. Figure 1 depicts the Preferred Reporting Items for Systematic Reviews and Meta-Analyses (PRISMA) flow diagram detailing the study selection process.

Therapies and updates

Iron supplementation has to be provided to all pregnant females, according to the recommendations made by the International Nutritional Anaemia Consultative Group, WHO, and the United Nations Children's Fund, because the requirements for iron during pregnancy are not easy to meet solely through diet. Supplementary intake should continue following delivery in regions where IDA incidence is greater than 40% [4]. A balanced diet and iron supplements are used to deal with iron deficiency. As a prophylactic intervention, iron supplementation throughout the pregnancy may improve the mother's haematological status and birth weight. WHO has long advocated using iron supplements during pregnancy in low- and middle-income countries and many high-income countries [13]. Recently, an iron and folic acid supplementation regimen for preventing anaemia in pregnant females has been advocated, with specific properties depending on the target population to be treated [14]. However, the primary problem is associated with the high cost of these treatment modalities and diagnostic methods. Researchers and international organisations should work together to improve anaemia management globally by lowering the prices of diagnostic methods in developing countries, discovering modern indicators for iron deficiency cases, and uncovering other relationships between iron deficiency and feto-maternal adverse effects.

Promoting proper nutrition is vital, especially among pregnant women in rural and marginalised communities. Programs emphasising the importance of a balanced diet rich in iron and other essential nutrients can significantly reduce the prevalence of low Hb levels. The optimal treatment involves oral iron therapy with gradual restoration of iron content and Hb level maintenance. The bivalent ferrous or trivalent ferric form may be incorporated into the formulations, with the bivalent preparations being more readily absorbed than the trivalent formulations. Another study found that ferric carboxymaltose helped treat iron-deficient anaemia, boosting Hb levels and enhancing iron reserves while remaining tolerable [15].

Folic acid constitutes a vitamin of the B group required for DNA synthesis and neural tube development. The fetus's folate need rises throughout pregnancy, and lacking this nutrient might result in megaloblastic anaemia [16]. As a result, a new iron and folic acid supplementation programme has been developed to prevent anaemia in pregnancy, with varied features depending on the population to be treated. Improved haematological status during the gestational period may also lower the risk of mortality in females suffering from antepartum and obstetric haemorrhage and contribute to improved iron status in the postpartum period (six weeks after delivery) [17]. For almost 40 years, oral iron has been a first-line modality for treating IDA in pregnancy across India. Nevertheless, adverse effects, low adherence to oral medication consumption, and little medicinal efficiency are among the reasons why a new standard for IDA treatment regimens in pregnant females is being considered [18]. Table 1 summarises the differences between oral and parenteral iron therapy [19,20].

**Table 1:** Differences between oral and parenteral iron therapy* *[19,20] IDA: Iron deficiency anaemia

Sr. no.	Oral iron therapy	Parenteral iron therapy
1	Cost-effective and easy	Cost-ineffective and difficult
2	Self-administration	Requires hospital settings for administration
3	Takes time to replenish iron stores	Replenishes iron stores better and faster than oral therapy
4	Post-lactational anaemia is seen in the majority of cases	Post-lactational anaemia is prevented
5	Gastrointestinal side effects are predominantly seen	Gastrointestinal side effects are fewer as compared to oral iron therapy
6	Not suitable for lower haemoglobin values in the last trimester as the absolute increase in haemoglobin values is low	Very effective in the treatment of IDA in the third trimester as the absolute increase in haemoglobin values is higher
7	Weight-dependent formula for dosage is used	The dose depends on the degree of deficiency

Newer interventions

India has the highest anaemia rates worldwide, with the National Family Health Survey indicating that anaemia affects more than 50% of pregnant women across the country [21]. In India, the Anaemia Mukt Bharat-Intensified National Iron Plus Initiative aims at lowering the anaemia preponderance within females of childbearing age, adolescents, and children by 3% per year, thereby facilitating the attainment of a global World Health Assembly 2025 goal of achieving a 50% decline in anaemia among women of childbearing age [21]. In the same study, over 60% of children aged between 6 and 59 months were classified as deficient in iron with a Hb value of 11 g/dL due to anaemia during pregnancy. Adequate iron is needed for proper fetal, neonatal, and childhood brain development [22,23]. On the other hand, women who have moderate to severe anaemia in the second and third trimesters of gestation usually cannot compensate for the associated iron shortage. Thus, in spite of active transport through the placenta, less amount of iron may be supplied to the growing fetus, potentially leading to future neurodevelopmental impairment in the newborn [24].

Community-based programs involve engaging communities through awareness campaigns and involving local leaders can be instrumental in combating maternal anaemia. Community healthcare professionals can play an important role in spreading awareness and ensuring pregnant women receive appropriate care. The Government of India has declared reducing anaemia incidence in India a key priority, and several programmes have been implemented to accomplish this objective. To make the treatment financially feasible in the lower socioeconomic groups, various cash transfer programs and initiatives involving frequent, low-cost antenatal check-ups are introduced. Table 2 details the standard health programmes introduced to reduce MMR in India.

**Table 2:** Major health programmes and their key features ANC: antenatal care; ASHA: accredited social health activist; INR: Indian rupee

Name of the programme	Launch date	Governing body	Initiatives
Anaemia Mukt Bharat [25]	March 2018	Ministry of Health and Family Welfare	Targets at boosting current mechanisms and developing anaemia-prevention solutions. Follows 6x6x6 strategy. Includes 6 beneficiaries, 6 interventional strategies as well as 6 institutionalised mechanisms
Pradhan Mantri Surakshit Matritva Abhiyan (PMSMA) [26]	9 June 2016	Ministry of Health and Family Welfare	Offers free-of-charge ANC check-ups on the 9th of every month. In addition to this, the extended PMSMA for high-risk pregnancies provides 3 extra ANC visits, more incentives to ASHA, and free transportation of beneficiaries
Janani Suraksha Yojna (JSY) [27]	12 April 2005	Ministry of Health and Family Welfare	Incentives are given to both mothers and ASHA workers to promote institutional delivery. Based on rural or urban populations in high-performing and low-performing states, incentives are different
Pradhan Mantri Matru Vandana Yojana (PMMVY) [28]	1 September 2017	Ministry of Women and Child Development	INR 5000 is provided to the mother in 3 installments. INR 1000 at early registration of pregnancy, INR 2000 after the first ANC visit, and INR 2000 after registration of childbirth, and the child should receive the first cycle of immunisation
Janani Shishu Suraksha Karyakram (JSSK) [29]	1 June 2011	Ministry of Health and Family Welfare	Provides free transport; drugs including iron and folic acid supplements. Free delivery and free blood transfusion if needed to registered ANC cases
Surakshit Matritva Aashwasan (SUMAN) [30]	10 October 2019	Ministry of Health and Family Welfare	Scheme targeting zero preventable maternal and newborn fatalities. Provides dignified and respectful care to mothers

Anaemia Mukt Bharat

Anaemia Mukt Bharat is a multi-faceted initiative that aims to tackle the pervasive issue of anaemia in India by focusing on early detection, supplementation, nutrition education, and healthcare infrastructure improvement. Its ultimate goal is to create a healthier and anaemia-free future for the people of India, particularly vulnerable populations like pregnant women and children. The comprehensive strategy remains the same, but specific interventional guidelines have been revised and they are now made accessible online. The nationwide aim of the Anaemia Mukt Bharat campaign is to cut down anaemia preponderance among children, adolescents, and women of reproductive age by 3% per year. It also plans to promote the achievement of a 2012 Global World Health Assembly target of attaining a fall in anaemia cases by 50% among women of reproductive age by the year 2025 [31]. 

MAMATA Conditional Cash Transfer Programme

Conditional cash transfers (CCTs) are demand-side interventions that involve cash transfers to fulfill health-enhancing factors like regular health check-ups and visits and human capital investment. Odisha, an Indian state, started the MAMATA Scheme in 2011, a statewide CCT scheme targeted to improve maternal/child health outcomes and increase health-adapting behaviours. MAMATA focuses on pregnant and breastfeeding females and gives a substantial monetary incentive compared to household income levels. MAMATA and other CCT programmes have had a favourable influence on maternal and child wellbeing indices. They have the potential to boost the effect of maternal healthcare services, reduce maternal deaths, and improve the likelihood of neonates getting critical treatment shortly after birth [32].

Pradhan Mantri Surakshit Matritva Abhiyan

On June 9, 2016, the Government of India's Ministry of Health and Family Welfare launched the Pradhan Mantri Surakshit Matritva Abhiyan (PMSMA). The plan aims to provide free-of-charge, assured, thorough, and prompt prenatal care to all pregnant females on the 9th day of each month. This initiative may prove to be a boon to tackle high-risk pregnancies in the early period of gestation itself. High-risk pregnancies account for 20-30% of all pregnancies and 75% of all perinatal morbidity and death in India. However, only 14% of pregnancies are now classed as 'High Risk' on the PMSMA reporting platform. A recent update has extended PMSMA for tracking high-risk pregnancies to detect missed cases of high-risk pregnancies. As per that scheme, states can organise an additional day for PMSMA clinics (in addition to the current 9th day of every month); once a pregnancy is classified as high risk (HRP), the particular accredited social health activist (ASHA)/ANM must arrange for three further ANC visits by a doctor/obstetrician for that HRP. ASHAs are now rewarded with more incentives for mobilizing HRP beneficiaries for follow-up visits to the nearest PMSMA clinics/healthcare facilities; beneficiaries will benefit from free transportation during PMSMA sessions. During the second and third trimesters of gestation, a minimal package of prenatal care facilities is offered to beneficiaries at PMSMA healthcare centres to guarantee that each pregnant female receives at least one antenatal visit from a physician/specialist at designated government health facilities [26,33].

Janani Suraksha Yojana

The Janani Suraksha Yojana (JSY) is the most extensive conditional money distribution programme. Its principal purpose is to promote facility-based delivery in India to decline maternal and newborn mortality. The JSY has also led to a decrease in out-of-pocket delivery care expenditures and an increase in antenatal care [34]. The performing status of states decides the incentives given to the mother and ASHA workers. Incentives are higher in the states with low performance, such as Bihar, Uttar Pradesh, Madhya Pradesh, Jharkhand, Rajasthan, Chattisgarh, Odissa, and Assam along with Jammu and Kashmir, where the incentives given to mother and ASHA workers amount to INR 1400 and 600 respectively in rural areas and INR 1000 and 400 INR in urban localities. At the same time, the incentives given in high-performing states amount to INR 700 and 600 to mothers and ASHA, respectively, in rural populations and INR 600 and 400 in urban regions [34].

Food Fortification Using Iron

Regardless of the acknowledged advantages of prenatal folic acid and iron supplements as well as multiple micronutrient supplementation, low adherence to regular therapy has limited the intervention's efficacy in many cases. Supplemental micronutrient powders for fortification are single-dose mineral and vitamin sachets that may be sprinkled on cooked foods to boost their nutritional value. Using several micronutrient powders for the fortification of food items in pregnant females may be an alternative to vitamin supplementation in the gestational period [35]. Ensuring the consistent distribution of iron in fortified foods, addressing customer acceptance and taste concerns, and monitoring and enforcing compliance among food makers are all challenges associated with food fortification. By boosting the iron content of widely consumed foods, dietary fortification with iron is an effective technique for combating iron deficiency anaemia and improving general health. It is a cost-effective and long-term strategy for increasing iron consumption, particularly in populations at risk of iron insufficiency [35].

Table 3 provides a summary of all the articles analyzed in this review article.

**Table 3:** Summary of studies MMR: maternal mortality rate; UN: United Nations; MDG: Millennium Development Goals; IFA: iron-folic acid; NTD: neural tube defect; IDA: iron deficiency anaemia; ANC: antenatal care; JSY: Janani Suraksha Yojana; PMMVY: Pradhan Mantri Matru Vandana Yojana; IGMSY: Indira Gandhi Matritva Sahyog Yojana; JSSK: Janani Shishu Suraksha Karyakram; CCT: conditional cash transfer; MNP: multiple micronutrient powders

Author, year of publication	Title	Observations
Means, 2020 [1]	Iron deficiency and iron deficiency anemia: implications and impact in pregnancy, fetal development, and early childhood parameters	Iron deficiency in pregnancy can lead to fatigue, weakness, and an increased risk of infections. IDA, a more severe form of the condition, can further exacerbate these symptoms. Iron is crucial for fetal development, particularly in the second and third trimesters when the fetus rapidly accumulates iron. Iron deficiency during this period can result in impaired fetal growth and development. Iron is essential for the development of the infant's brain. Iron deficiency during early childhood can lead to cognitive deficits, impaired motor skills, and behavioral problems
Raut and Hiwale, 2022 [2]	Iron deficiency anemia in pregnancy	Suggests an approach to diagnose and manage IDA in pregnancy
Sifakis and Pharmakides, 2006 [3]	Anemia in pregnancy	IDA and megaloblastic anaemia are the most prevalent anaemias during pregnancy, and they are more likely in women who have poor diets and do not receive prenatal iron and folate supplementation
Roy and Pavord, 2018 [4]	The management of anaemia and haematinic deficiencies in pregnancy and post-partum	A plan for iron replacement in an anaemic pregnant woman population must be devised not only based on what is physiologically and clinically most suitable, but also in the context of each organisation's delivery of care structure, taking cost-effectiveness into account. For that, management algorithms must be regionally tailored to ensure they match basic clinical imperatives
Benson et al., 2020 [5]	Iron deficiency anaemia in pregnancy: a contemporary review	Intravenous iron looks to be a safe therapy for rapidly correcting maternal anaemia, but further study on patient outcomes and cost-effectiveness is needed
Khalafallah and Dennis, 2012 [6]	Iron deficiency anaemia in pregnancy and postpartum: pathophysiology and effect of oral versus intravenous iron therapy	In some clinical settings, intravenous iron should be explored as an effective, quick, and safe therapeutic alternative
Hill et al., 2007 [7]	Estimates of maternal mortality worldwide between 1990 and 2005: an assessment of available data	While some regions have made headway in lowering maternal fatalities since 1990, maternal mortality ratios in sub-Saharan Africa have remained extremely high
Prakash et al., 1991 [8]	Maternal mortality in India: current status and strategies for reduction.	The low quality of maternal healthcare in India is attributed to insufficient coordination between delivery system levels and treatment fragmentation. This article also provides effective strategies for reducing MMR
Parks et al., 2018 [9]	Maternal anaemia and maternal, fetal, and neonatal outcomes in a prospective cohort study in India and Pakistan.	Severe maternal anaemia is linked to an increased risk of poor maternal, fetal, and neonatal outcomes, although milder levels of anaemia are not. Preventive interventions for severe anaemia in pregnant women should be studied
Akseer et al., 2017 [10]	Progress in maternal and child health: how has South Asia fared?	Improvement in the present level of reproductive, maternal, neonatal, and child health in South Asia compared to research conducted 12 years ago by Nakseer et al.
Meh et al., 2021 [11]	Trends in maternal mortality in India over two decades in nationally representative surveys.	India could achieve the UN 2030 MMR goals if the average rate of reduction is maintained. However, without further interventions, the poorer states will not
Shah et al., 2014 [12]	Changing epidemiology of maternal mortality in rural India: time to reset strategies for MDG-5.	It is essential to prioritise the care of indirect causes of maternal mortality during pregnancy at community and hospital levels to attain MDG-5
Osungbade and Oladunjoye, 2012 [13]	Preventive treatments of iron deficiency anaemia in pregnancy: a review of their effectiveness and implications for health system strengthening.	Except for the stated limits, prophylactic iron supplementation and dietary fortification with iron can improve mother and child health. Sustained lobbying at the national and international policy levels to address micronutrient deficiencies is also required to achieve Millennium Development Goals 4 and 5
Chatterjee et al., 2016 [14]	Iron supplement use in pregnancy - are the right women taking the right amount?	Women consume variable amounts of iron, and certain high-risk women are given insufficient amounts to prevent or cure iron deficiency. Healthcare experts are best placed to advise women on the use of iron supplements during pregnancy, and they should educate women individually on the type and amount of supplement most suited to their requirements
Varghese et al., 2019 [15]	Demand and supply factors of iron-folic acid supplementation and its association with anaemia in North Indian pregnant women.	Anaemia among pregnant Indian women remains a priority for the government; targeted interventions such as regular haemoglobin testing and ensuring successful implementation of the National Iron Plus Initiative nuanced for the local context and access to IFA can help India meet anaemia reduction targets
Greenberg et al., 2011 [16]	Folic acid supplementation and pregnancy: more than just neural tube defect prevention.	Folic acid supplementation during pregnancy protects against fetal structural malformations such as NTD and congenital heart problems. According to recent research, it may help protect against premature delivery. The significance of genetic variants in the genes governing folate metabolism (especially the MTHFR gene) and how they impact l-methylfolate bioavailability and, hence, folate supplementation techniques, is not well known
Lewkowitz and Tuuli, 2019 [17]	Iron-deficiency anaemia in pregnancy: the role of hepcidin.	Understanding the role of hepcidin in iron metabolism during pregnancy is important for developing effective strategies to prevent and treat IDA. It may involve interventions such as dietary iron supplementation, iron-rich foods, or medications that can modulate hepcidin levels to ensure adequate iron absorption and utilisation during pregnancy
Tandon et al., 2018 [18]	Management of iron deficiency anemia in pregnancy in India.	This study proposes an algorithm for diagnosing and managing IDA in pregnancy based on anaemia severity and gestational age that is suitable for general use in resource-limited settings. We also offer measures to raise public awareness and address this health concern, such as observing "National Anaemia Awareness and Treatment Day"
Bayoumeu et al., 2022 [19]	Iron therapy in iron deficiency anemia in pregnancy: intravenous route versus oral route.	Iron sucrose appears to be a treatment without serious side effects indicated in the correction of pregnancy anaemia or iron store depletion
Shi et al., 2015 [20]	Intravenous iron sucrose versus oral iron in the treatment of pregnancy with iron deficiency anaemia: a systematic review.	Intravenous iron sucrose was related to fewer adverse events and was more effective than normal oral iron therapy in pregnant women who could not tolerate the adverse effects of oral treatment or required a quick restoration of iron reserves
Derman et al., 2021 [21]	RAPIDIRON: reducing anaemia in pregnancy in India-a 3-arm, randomized-controlled trial comparing the effectiveness of oral iron with single-dose intravenous iron in the treatment of iron deficiency anaemia in pregnant women and reducing low birth weight deliveries.	The RAPIDIRON study shows that a single-dose intravenous iron infusion is more efficacious and cost-efficient than the current standard of treatment in lowering IDA in pregnancy
Juul et al., 2019 [22]	Perinatal iron deficiency: implications for mothers and infants.	Screen all pregnant women for iron deficiency regardless of hemoglobin levels when they appear for prenatal care. Administer iron supplements if the iron deficiency is present with or without anaemia. If iron deficiency is present in the first trimester, prescribe oral iron (we do not advocate intravenous iron in the first trimester owing to a lack of safety evidence), and if iron deficiency continues, intravenous iron should be seriously considered once the pregnancy enters the second trimester. If oral iron is given, we recommend taking one pill every other day. All high-risk neonates (including preterm newborns, diabetic mothers' infants, anaemic or iron-deficient mothers' infants, and smokers' infants) should be checked for iron deficiency at delivery
Juul, 2012 [23]	Erythropoiesis and the approach to anemia in premature infants.	Clinical practises can have a significant influence on anaemia in preterm babies. Delaying cord clamping, reducing phlebotomy loss, and optimising nutritional assistance are all practices that can help to reduce the severity of anaemia and hence the requirement for transfusions or erythropoietin therapy
Georgieff et al., 2018 [24]	Atypical fetal development: fetal alcohol syndrome, nutritional deprivation, teratogens, and risk for neurodevelopmental disorders and psychopathology.	This article contains evidence to support the concept that similar fetal experiences influence brain development and function over the life span via several pathways
Kinjawadekar, 2023 [25]	Aiding the vision of an ‘Anemia Mukt Bharat’.	Describes Anaemia Mukt Bharat scheme
Dandona et al., 2022 [26]	Assessment of quality of antenatal care services in public sector facilities in India.	Grossly inadequate quality of ANC services under the Pradhan Mantri Surakshit Matritva Abhiyan needs urgent attention to improve maternal and neonatal health outcomes
Rai and Singh, 2012 [27]	Janani Suraksha Yojana: the conditional cash transfer scheme to reduce maternal mortality in India - a need for reassessment.	The Government of India started a countrywide conditional cash transfer (CCT) initiative named Janani Suraksha Yojana (JSY) in 2005 under the umbrella of the countrywide Rural Health Mission to encourage women to give birth in health facilities, thereby reducing maternal fatalities
Von Haaren and Klonner, 2021 [28]	Lessons learned? intended and unintended effects of India’s second-generation maternal cash transfer scheme.	The only significant distinction between PMMVY and IGMSY is that PMMVY only covers a woman's first birth, whereas IGMSY covers the first two deliveries
Tripathi et al., 2022 [29]	Impact of Janani Shishu Suraksha Karyakram on out-of-pocket expenditure among urban slum dwellers in Northern India.	Strengthening of implementation of JSSK is required to ensure universal access to natal care
Prajapati et al., 2022 [30]	Prevalence of high-risk pregnancy among pregnant women enrolled under Pradhan Mantri Surakshit Matritva Abhiyan in government health facilities of district Etawah, Uttar Pradesh: a cross-sectional study.	To minimise maternal mortality, regular ANC check-ups, early detection of high-risk pregnancies, health education, and prompt screening are required
Ahmad et al., 2023 [31]	Public health supply chain for iron and folic acid supplementation in India: status, bottlenecks and an agenda for corrective action under Anemia Mukt Bharat strategy.	The Government of India started a countrywide CCT initiative named Janani Suraksha Yojana (JSY) in 2005 under the umbrella of the countrywide Rural Health Mission to encourage women to give birth in health facilities, thereby reducing maternal fatalities
Chakrabarti et al., 2021 [32]	Maternal and child health benefits of the Mamata Conditional Cash Transfer Program in Odisha, India.	This study demonstrates the feasibility of prospective improvements in maternal and child nutrition outcomes following CCTs that reward healthcare use in India
Bhatia et al., 2021 [33]	Pro-poor policies and improvements in maternal health outcomes in India.	One of the critical reasons for the success of many current initiatives has been community-level engagement, with community health workers playing a vital role. Policymakers must prioritise underperforming states and socioeconomic groups within states by concurrently addressing demand-side and supply-side policies as mediated by contextual variables
Sen et al., 2020 [34]	Unintended effects of Janani Suraksha Yojana on maternal care in India.	Receiving economic help for institutional birth through the cash transfer initiative resulted in higher future usage of contraception, breastfeeding initiation, and postnatal check-ups. Aside from upgrading current health facilities focusing on low-performing states, continuing the conditional cash transfer scheme for maternity care is strongly recommended
Suchdev et al., 2015 [35]	Multiple micronutrient powders for home (point-of-use) fortification of foods in pregnant women.	MNPs have been studied as a potential intervention to address nutrient deficiencies in pregnant women, with the aim of improving maternal and fetal health outcomes. It is important to note that the effectiveness and safety of MNPs can depend on various factors, including the specific formulation of the product, the nutritional status of pregnant women, and the overall quality of ANC

## Conclusions

Anaemia is a leading cause of impairment globally, making it a significant global public health challenge. The primary goal of this article is to create awareness about anaemia in pregnancy. The authors examined the literature on anaemia during pregnancy, offering current and unambiguous guidance for preventing and managing this illness, which, if not appropriately managed, can result in severe maternal and neonatal issues. These articles and suggestions were published in the last 10 years and provide accurate information about this ailment. We incorporated a few earlier publications that we deemed appropriate for effectively describing the condition. Maternal anaemia is a treatable illness and India has made tremendous progress in lowering its prevalence and accompanying maternal death rates. India can maintain its gains in ensuring healthier pregnancies and lowering maternal mortality through initiatives such as providing supplements, improved nutrition, prenatal care, and community participation. Government agencies, healthcare providers, and communities must collaborate to prioritise maternal health and successfully address maternal anaemia. The Indian government has introduced various new programmes and treatment options that may aid in preventing anaemia and providing better antenatal care. Interventions that promote frequent antenatal visits, iron supplementation, iron-fortified foods, and financial help to pregnant women may aid in lowering maternal mortality due to anaemia.
